# Cutaneous Fistula Caused by Metallosis Following Rigid Spinal Fixation: A Rare but Important Diagnostic Consideration

**DOI:** 10.7759/cureus.83791

**Published:** 2025-05-09

**Authors:** Gen Yamauchi, Yu Yamamoto, Yusuke Nishimura, Masakazu Takayasu, Ryuta Saito

**Affiliations:** 1 Neurological Surgery, Kasugai Municipal Hospital, Kasugai, JPN; 2 Neurological Surgery, Inazawa Municipal Hospital, Inazawa, JPN; 3 Neurological Surgery, Nagoya University Hospital, Nagoya, JPN

**Keywords:** cutaneous fistula, instrument failure, metallosis, pedicle screw, spinal fixation, spine instrumentation

## Abstract

Metallosis is a rare complication of spinal instrumentation, typically associated with joint arthroplasty or fusionless constructs. Fistula formation due to metallosis is exceptionally uncommon, particularly in cases involving rigid spinal fixation. We report a case of a 48-year-old female patient who developed a cutaneous fistula at the site of a previous thoracic spinal fixation performed for a metastatic spinal tumor. Clinical examination revealed a small granulomatous mass with a fistula. Although dynamic radiographs showed no apparent instability, computed tomography revealed radiolucent halos around the screws in the right T4 and left T3 pedicles, indicating screw loosening. Magnetic resonance imaging demonstrated a sinus tract extending from the skin to the right T3 pedicle screw. Surgical excision of the fistula and hardware revision were performed. Histological analysis confirmed the presence of metal wear debris and a chronic inflammatory response consistent with metallosis. Bacterial cultures were negative. Despite the absence of typical intraoperative signs such as tissue discoloration or necrosis, metallosis was diagnosed pathologically. This case highlights the importance of considering metallosis in the differential diagnosis of unexplained cutaneous lesions overlying spinal instrumentation. Even in the absence of instability or typical intraoperative findings, metallosis may present solely with skin manifestations such as fistula formation. Early recognition is essential for appropriate management.

## Introduction

An increasing number of spinal instrumented fusion procedures are widely utilized for treating spinal disorders [1]. Metallosis is a condition characterized by the deposition of metallic particles in tissues originating from implanted foreign materials, and is accompanied by an inflammatory host response [2]. Metallosis is more commonly associated with joint replacement surgeries and is rarely seen in spinal surgeries. Among spinal cases, most have been linked to fusionless instrumentation systems used for idiopathic scoliosis treatment [3], while reports related to posterolateral rigid fixation surgery remain limited [4].

The clinical presentation of spinal metallosis is nonspecific, making its diagnosis reliant on clinical suspicion. A literature review on metallosis reported that 37% of cases, including asymptomatic ones, were incidentally identified [4]. Although late-onset postoperative pain is a common manifestation, it is often linked to complications such as pseudarthrosis, instrumentation loosening, or subacute infection [2]. Less frequently, inflammatory granulation tissue or metalloma formation can result in neural compression [2,4].

Metallosis associated with fistula formation is extremely rare. To our knowledge, this is the first case report of metallosis manifesting as a cutaneous fistula following rigid spinal fixation, which was diagnosed based solely on cutaneous findings [4].

## Case presentation

**Figure 1:**
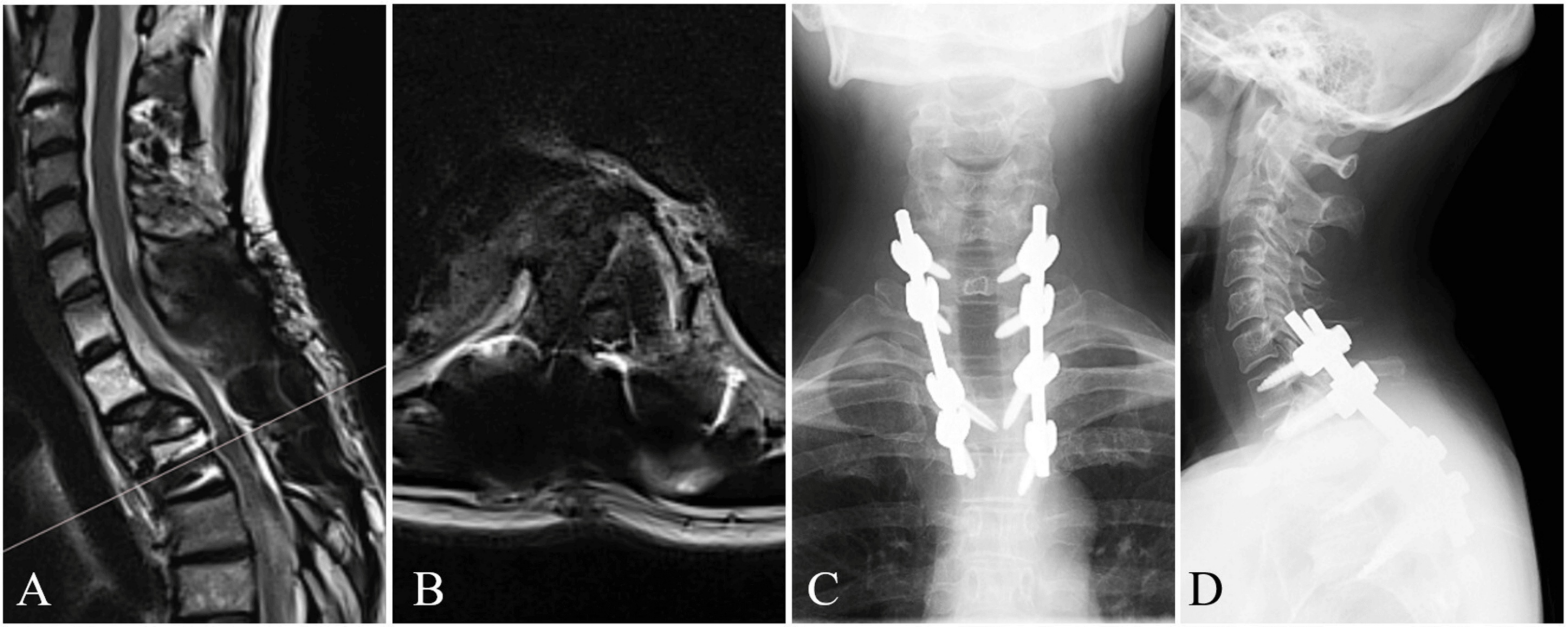
Magnetic resonance imaging and plain radiographs (A) T2-weighted sagittal image and (B) T2-weighted axial image at T3 obtained six months before presentation showed no abnormalities at the surgical site and no signs of seroma. (C, D) Plain radiographs at presentation. The spinal alignment was preserved and no radiographic evidence of instability was observed.

A 48-year-old female patient, previously treated with spinal fixation for thoracic metastasis from breast cancer, presented with a complaint of a lesion at the site of her surgical scar. She had undergone right mastectomy for breast cancer eight years earlier. Eighteen months before presentation, she had received 30 Gy of radiotherapy to a metastatic lesion at the T2 vertebra. One month after radiotherapy, the T2 vertebral body collapsed, and she underwent posterior decompression and fixation surgery. She made a good recovery postoperatively without any wound-related complications (Figure 1).

**Figure 2:**
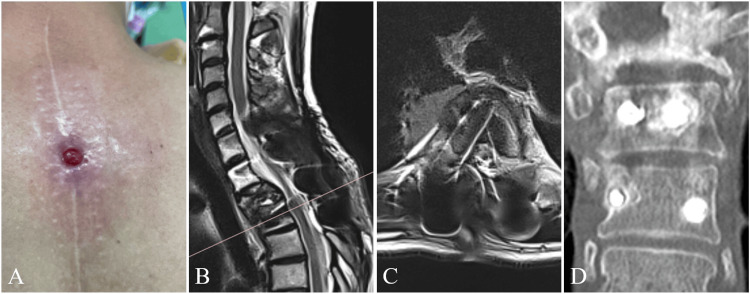
Cutaneous findings and radiological findings at the time of presentation MRI demonstrated a sinus tract (fistula) with high signal intensity on T2-weighted images, extending from the cutaneous lesion to the right T3 pedicle screw. CT images revealed a slight radiolucent halo around the screw in the right T4 and left T3 pedicles. (A) Cutaneous appearance, (B) T2-weighted sagittal image, (C) T2-weighted axial image, (D) CT coronal image. MRI: magnetic resonance imaging, CT: computed tomography

At presentation, a healing granulomatous mass measuring 7 mm in diameter was observed on the surgical scar, accompanied by mild erythema and a central ulceration. Additionally, a cutaneous fistula opening into the subcutaneous tissue was noted adjacent to the mass. There was a slight amount of serous exudate though no signs of overt infection were present (Figure 2).

Plain lateral radiographs (in flexion and extension) showed no apparent instability or screw loosening. CT images revealed a slight radiolucent halo around the screws in the right T4 and left T3 pedicles. MRI demonstrated a sinus tract (fistula) with high signal intensity on the T2-weighted images and low signal intensity on the T1-weighted images, extending from the cutaneous lesion to the right T3 pedicle screw (Figure 2). A granulomatous skin mass with a sinus tract connecting to the implant strongly suggested a foreign body reaction rather than a surgical site infection. Finally, a diagnosis of fistula formation due to metallosis associated with the spinal implant was made.

Surgical procedure

**Figure 3:**
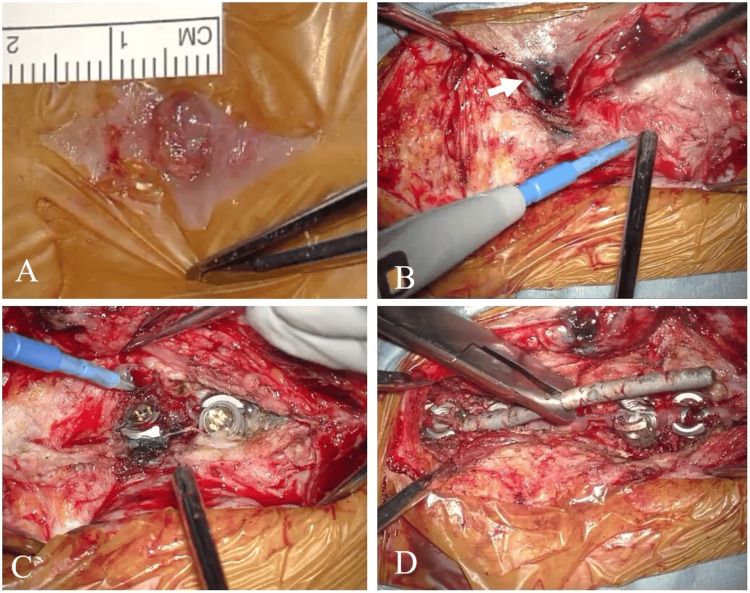
Intraoperative findings (A) A granulomatous mass (7 mm) was identified. (B) Resection was performed with care to minimize exposure of the stained fistula tract. (C, D) Although the T3 screw head area was stained, no discoloration or necrotic tissue characteristic of metallosis was observed.

The surgery involved an excision of the cutaneous fistula, removal of the right T3 pedicle screw (which was connected to the fistula), and caudal extension of spinal fixation by one vertebral level (Figure 3).

Indigo carmine was injected into the fistula to stain the sinus tract. The granulomatous lesion on the skin surface was excised. To prevent contamination of the surgical field with superficial flora, the stained tract was carefully excised without excessive exposure. The indigo-stained tract extended to the head of the T3 pedicle screw without spreading beyond that point. All the stained tissue was successfully resected en bloc. There was no obvious loosening between the rod and the screw head. The T3 pedicle screw, which was connected to the fistula, was removed. A new pedicle screw was inserted into the right T5 pedicle with the fixation level extended caudally up to T5. Samples from the deep portion of the fistula and from around the implant were submitted for bacterial culture and histopathological analysis.

Histopathological findings of the deep fistula

**Figure 4:**
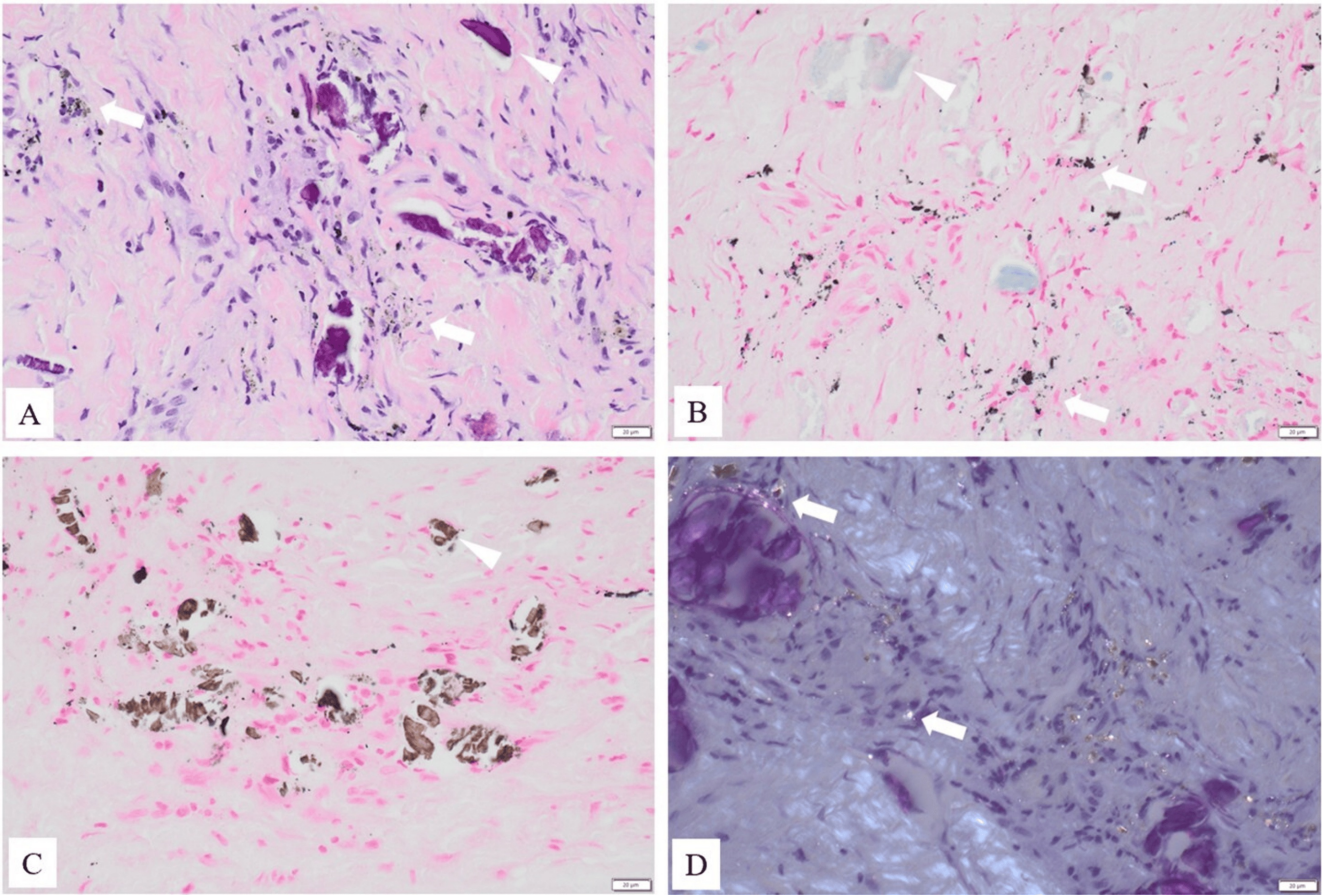
Histopathological findings of the deep fistula (A) Hematoxylin and eosin stain: Extensive granulation tissue was observed, characterized by proliferation of collagen fibers and neovascularization. Numerous fine particles suspected to be foreign material were scattered within the granulation tissue (white arrow), and some of these were phagocytosed by macrophages. Bone fragments were also identified (arrowhead). (B) Bleaching and Berlin blue stain: The particles were resistant to bleaching and showed negative staining with Berlin blue. (C) Von Kossa stain: Negative. The arrowhead indicates a bone fragment. (D) Polarized light microscopy: The particles exhibited marked birefringence (white spots, white arrow). Given that the implanted device used during surgery was made of titanium alloy, these particles were diagnosed as metal wear debris derived from the titanium alloy. The scale bar in the lower right corner indicates 20 μm.

**Figure 5:**
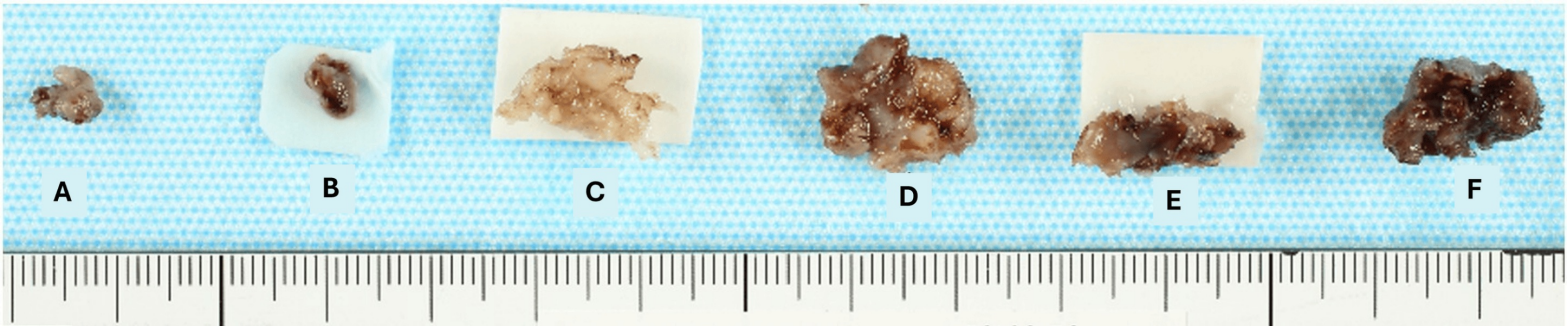
Gross photograph of the resected fistula (A) Granulomatous mass; (B) Superficial portion of the fistula; (C) Scar tissue surrounding the T4 screw head; (D) Scar tissue surrounding the T3 screw head; (E) Scar tissue around the rod; (F) Deep portion of the fistula

The details of the histopathological findings and the resected fistula are shown in Figures 4, 5.

Bacterial culture

Bacterial cultures of deep fistula tissue and periprosthetic samples were all negative.

Postoperative diagnosis

Histological examination of the deep fistula tissue revealed metal particles and the surrounding inflammatory response, leading to the diagnosis of a cutaneous fistula caused by metallosis.

Postoperative course

On postoperative day 40, the patient developed a superficial surgical site infection, which was successfully treated by local wound revision and debridement. No recurrence of the fistula has been observed during the three-year follow-up period.

## Discussion

We report the first case of cutaneous fistula formation caused by metallosis following spinal fixation surgery. Early diagnosis and prompt treatment of this rare manifestation are essential.

Metallosis is believed to result from a combination of abnormal micromotion and a continuous inflammatory, chemically mediated reaction [4]. Mechanical phenomena (e.g., wear), chemical processes (corrosion), electrochemical reactions (galvanic corrosion), and biological responses may all contribute to the pathology to varying degrees [2,4,5]. Initially, micromotion at the bone-metal or metal-metal interface leads to metallic corrosion and the release of metal wear particles. These particles then trigger an inflammatory foreign body response, including macrophage migration. Phagocytosis of the particles initiates a further cascade of inflammatory reactions, which in turn accelerate implant degradation and can lead to loosening, bone resorption, or granuloma formation [2,6,7]. If the abnormal micromotion and subsequent responses persist, metallosis may eventually manifest clinically.

Although metallosis has no specific clinical presentation and may be entirely asymptomatic, some patients reportedly demonstrated low back pain, neurological deficits such as motor weakness due to granuloma, and radiographic complications such as loosening, pseudarthrosis, and progression of spinal deformity [2,4,5,7]. While several reports of metallosis have involved expandable systems in spinal surgeries, rigid spinal fixation systems are rarely associated with metallosis, particularly fistula formation [3,4]. Radiological findings are also nonspecific, however, pseudotumoral images of mass-like lesions (i.e., metallomas) close to the fixation implants have been most likely observed [4]. Intraoperative findings commonly include black or dark fibrotic tissue, often accompanied by a collection of liquid [3,4]. The implant may appear loosened or displaced due to bone resorption. Ultimately, the diagnosis of metallosis requires pathological confirmation of metallic debris and an associated inflammatory response [8,9].

In our case, no characteristic intraoperative signs of metallosis were noted. There was no obvious discoloration of the surrounding tissues, and no apparent loosening between the screw head and the rod. However, a pathological analysis of periprosthetic tissue confirmed the presence of debris and inflammation, leading to a definitive diagnosis. Although a localized infection may contribute to the development of metallosis, all intraoperative cultures in this case were negative. Although not a typical feature of metallosis, fistula formation may result from rupture of a periprosthetic seroma extending to the skin surface [3]. Although no seroma was detected on MRI during the course of follow-up, the continuity of the sinus tract from the skin to the implant raised the suspicion of metallosis.

Treatment of metallosis must address both the clinical or radiological problems caused by the condition and prevent its further progression. For instance, if neurological compression occurs due to a metalloma, surgical excision is necessary. In cases with loosening, pseudarthrosis, or deformity progression, revision fixation may be required. Even when preoperative diagnosis is not established, intraoperative findings suggestive of metallosis should prompt extensive debridement of necrotic and discolored tissues to prevent persistent chronic inflammation [2,4], with bacterial cultures to rule out infection and guide appropriate antibiotic therapy if needed. Whether implant removal is necessary or not remains a matter of debate [2,4]. According to previous reports, the underlying issue may not be the presence of the implant itself, but rather inadequate stabilization. In such cases, revision of instruments with or without an extension of the fusion levels has been reported to yield good outcomes [2,4,5,10]. Necrotic tissue serving as a substrate for chronic inflammation must also be widely resected [4,10]. Removal of implants should be performed when bony fusion is achieved because chronic inflammation resulting from chemical corrosion, galvanic corrosion, or biological interactions may only be resolved by the removal of the offending implant [2,4].

Although reduced implant fixation strength due to bone metastasis may contribute to mechanical instability and potentially trigger metallosis, it remains unclear whether subsequent inflammatory and chemically mediated reactions are more likely to occur in patients with cancer due to oncologic factors. It is possible that oncologic conditions such as breast cancer may influence the pathogenesis of metallosis through chronic systemic inflammation or local tissue susceptibility. Previous studies have shown that cancer and its treatments, such as radiotherapy, can disrupt normal tissue integrity and impair wound healing, potentially increasing the risk of inflammatory complications, including foreign body reactions [11,12]. We believe that, in our case, a combination of prior radiotherapy, systemic cancer-related inflammatory dysregulation, and localized micromotion contributed to this rare presentation of metallosis manifesting as a cutaneous fistula. Further accumulation of similar case reports will be necessary to clarify these potential associations.

## Conclusions

We report a rare case of cutaneous fistula formation caused by metallosis following rigid spinal fixation. Although metallosis is often asymptomatic or presents with nonspecific symptoms, it should be considered in the differential diagnosis of atypical skin lesions overlying spinal instrumentation. In this case, the diagnosis was established despite the absence of characteristic intraoperative signs such as tissue discoloration or loosening, emphasizing the importance of pathological confirmation and clinical suspicion. Prompt recognition and appropriate surgical management, including excision of necrotic tissue, hardware revision, and pathological evaluation, are essential to prevent chronic inflammation and recurrence. Awareness of this rare but important complication can lead to timely diagnosis and improved patient outcomes.
